# Expression divergence of the *AGL6 *MADS domain transcription factor lineage after a core eudicot duplication suggests functional diversification

**DOI:** 10.1186/1471-2229-10-148

**Published:** 2010-07-15

**Authors:** Tom Viaene, Dries Vekemans, Annette Becker, Siegbert Melzer, Koen Geuten

**Affiliations:** 1 Laboratory of Plant Systematics, Institute of Botany and Microbiology, Kasteelpark Arenberg 31, PO Box 2437, B-3001 Leuven, Belgium; 2 Evolutionary Developmental Genetics Group, University of Bremen, Leobener Str., UFT, 28359 Bremen, Germany

## Abstract

**Background:**

Because of their known role as transcriptional regulators of key plant developmental processes, the diversification of MADS-box gene function is thought to be a major driving force in the developmental evolution of plants. Yet the function of some MADS-box gene subfamilies has remained elusive thus far. One such lineage, *AGL6*, has now been functionally characterized in three angiosperm species, but a phylogenetic framework for comparison of *AGL6 *gene function is currently missing.

**Results:**

Based on phylogenetic analyses of newly isolated and EST-based sequences, we describe the duplication history of the *AGL6 *subfamily in angiosperms. Our analyses provide support for four ancient duplications in the evolution of the *AGL6 *lineage: one at the base of core eudicots resulting in *euAGL6 *and *AGL6-like *gene clades, one during basal angiosperm diversification and two in monocot evolution. To investigate whether the spatial domains in which *AGL6 *genes function have diverged after duplication, we use quantitative Real Time PCR. We show that the core eudicot *AGL6-like *clade acquired expression in vegetative tissues, while its paralog *euAGL6 *remains predominantly confined to reproductive tissues.

**Conclusions:**

These and previous data lead us to propose that the *AGL6 *lineage in core eudicots, in addition to functions related to the expression in reproductive structures, may have acquired a function in developmental transitions of vegetative shoots.

## Background

The *AGAMOUS LIKE 6 *lineage of MIKC-type MADS-box transcription factors is rooted in a superclade with both *SEPALLATA*-like genes and *APETALA1/FRUITFUL*-like genes, though the exact relationships between these three lineages are somewhat unclear [1-6]. The function of *SEP*- and *AP1*-like genes is known to contribute to floral meristem specification and floral organ identity in angiosperm reproductive development [5,7-11]. Yet the function of *AGL6 *genes, the third lineage in this superclade, had not been functionally characterized until recently.

The genome of the genetic plant model *Arabidopsis thaliana *harbors two *AGL6 *genes: *AGL6 *after which the subfamily was named and its paralog *AGL13*. No knockout phenotype has been described for either one of these two paralogs, possibly due to genetic redundancy with each other and other factors [12-15]. Just recently, knockout phenotypes have been described in three other angiosperm species: the grasses rice (*Oryza*) and maize (*Zea*) and in *Petunia*, an asterid species [16-18]. In *Oryza*, Ohmori *et al. *[16] characterized the function of *MADS6 *where it regulates floral organ identity and floral meristem determinacy and was renamed *MOSAIC FLORAL ORGANS1 *(*MFO1*). *Mfo1 *florets develop abnormal paleas and lodicules, mixed organs and extra floral organs [16]. In *Zea, bearded ear*, a loss-of-function mutant for *AGL6 *demonstrates a similar role in floral organ development and floral meristem identity [18]. Finally in *Petunia*, the flower specific function of *PhAGL6 *in petal and anther development was revealed in double and triple mutants with *SEPALLATA*-like genes, indicating that *PhAGL6 *functions redundantly with closely related genes [17]. In agreement with this role confined to reproductive development, *PhAGL6 *expression was not observed during vegetative developmental stages [17]. So far, the knockout phenotypes studied suggest that *AGL6 *plays a redundant role in establishing the flower and its organs.

Members of the *AGL6 *lineage have been isolated from gymnosperms [19-21] and all major angiosperm clades [1]. Comparing expression patterns of these genes throughout these plant lineages suggests that expression of *AGL6 *in floral meristems has been conserved since angiosperm origin [14,21,22]. Yet in *Arabidopsis *and gymnosperms, additional expression in vegetative tissues has been observed [12,13,19,20,22,23]. A detailed reporter analysis of the regulatory elements of *Arabidopsis AGL13 *and *AGL6 *observed expression of *AGL13 *in the vasculature underlying the shoot apical meristem [14]. In addition, expression of *AGL6 *was recently detected by *in situ *hybridization in cauline leaf primordia and in cryptic bract regions in response to floral induction [15]. Similarly, gymnosperm homologs have shown to be expressed in vegetative tissues [19,20]. For example, Mouradov *et al. *[20] detected expression of an *AGL6*-homolog (*PrMADS3*) in the group of cells initiating needle primordia in vegetative buds, suggestive of non-reproductive functions of the pre-angiosperm *AGL6 *lineage.

Though often frought with pleotropic effects unrelated to the original function, heterologous overexpression of *AGL6 *genes from *Hyacinthus orientalis *[24], *Oncidium *Gower Ramsey [25], *Dendrocalamus latiflorus *[26] and *Picea abies *[2] in *Arabidopsis *or *Nicotiana *suggested several other possible functions for *AGL6 *genes: a role in the juvenile to adult transition in Norway spruce [2] or a role as regulator of floral transition in the studied monocot species [24-26]. The latter function was corroborated by the constitutive expression in *Arabidopsis *of *AGL6 *or of *AGL6*- EAR, a repressor domain [15]. However, this early flowering phenotype was not obtained when an activated form (*AGL6*-VP16) was expressed under the *AGL6 *promoter, leaving a putative function for the *AGL6 *gene in the regulation of *Arabidopsis *flowering time to be confirmed.

The long-awaited functional characterization of *AGL6 *now highlights the need for a phylogenetic framework to understand the origin and diversification of *AGL6 *gene functions. The evolutionary history of the *AGL6 *gene lineage has been addressed in detail for grasses indicating that paralogs of *AGL6 *in *Oryza sativa *originate from an ancient duplication before the origin of the grass clade [23]. Here we present an analysis of the phylogenetic history of *AGL6 *genes in angiosperms by extending the available *AGL6 *sequence sampling with newly isolated asterid and EST-based eudicot sequences. Our inferences indicate four previously unidentified ancient duplications: one at the base of core eudicots resulting in what we have named *AGL6-like *and *euAGL6 *gene clades, two in monocots and a fourth one in basal angiosperms. Tracing expression patterns along the phylogeny of the *AGL6 *lineage indicates that expression of paralogs after the major core eudicot duplication has diverged to include expression in vegetative tissues.

## Methods

### Cloning of *AGL6 *MADS-box genes

Floral buds from *Philadelphus pubescens *(Hydrangeaceae), *Alangium platinifolium *(Cornaceae), *Galax urceolata *(Diapensiaceae), *Diospyros digyna *(Ebenaceae), *Gustavia brasiliensis *(Lecythidaceae), *Roridula gorgonias *(Roridulaceae), *Saurauia zahlbruckneri, Actinidia chinensis *(Actinidiaceae), *Asarum europaeum *(Aristolochiaceae), *Papaver somniferum *(Papaveraceae), *Berberis julianae *(Berberidaceae), *Anemone nemorosa *(Ranunculaceae) and *Citrus sinensis *(Rutaceae) were frozen in liquid nitrogen and stored at -80°C. Total RNA was isolated using the Invisorb Spin Plant RNA kit (Invitek, Berlin, DE) or Trizol (Invitrogen, Carlsbad, US). The mRNA was reverse transcribed into cDNA using AMV reverse transcriptase (Promega, Madison, US) and an oligo-dT primer. Our initial strategy was applying 3'-RACE [27] using primers (AP1MDS3: 5'-GTICARYTIARRMGIATIGARAAYAAGAT-3', RQVT: 5'-CGRCARGTGACSTTCTSCAARCG-3', oligodT: 5'-CCGGATCCTCTAGAGCGGCCGC(T)17-3') and according PCR-programs taken from the literature [21,28]. All PCR amplifications were carried out using *Taq *DNA Polymerase (Invitrogen, Carlsbad, US). PCR products were gel-purified with a Nucleospin extract 2 kit (Macherey-Nagel, Düren, DE) and cloned into the pGEM-T vector (Promega, Madison, US). After transformation, between 50 and 100 white clones were checked for inserts in a PCR reaction using the same primers and program. Plasmid DNA for selected clones was extracted with the Nucleospin Plasmid kit (Macherey-Nagel, Düren, DE). The plasmid inserts were sequenced using T7 and SP6 universal primers using the BigDye Terminator 1.1 kit (Applied Biosystems, Forster City, US) on an Applied Biosystems 310 sequencer or the plasmids were sent for sequencing (MacroGen Inc., Seoul, KP). *AGL6*-homologs from *Asarum europaeum *(*AeAGL6 *- 293 bp), *Anemone nemorosa *(*AnAGL6 *- 199 bp), *Berberis julianae *(*BjAGL6 *- 199 bp), *Papaver somniferum *(*PasAGL6 *- 199 bp) and *Citrus sinensis *(*CsAGL62 *- 98 bp) were cloned using a specific primer combination based on sequences of closely related species. In total, sixteen new *AGL6 *sequences were deposited in Genbank [Accession numbers HM121967 - HM121982]. In addition, *SEPALLATA3*-homologs were amplified from *Asarum europaeum *(*AeSEP3 *- 209 bp), *Papaver somniferum *(*PsSEP3 *- 420 bp), *Berberis juliana *(*BSEP3 *- 420 bp) and *Anemone nemorosa *(*AnSEP3 *- 420 bp) using specific primers from closely related species. New *SEP3*-sequences were deposited in Genbank [Accession numbers HM121963 - HM121966]. For all used primers see additional File 1: List of primer sequences used.

### Sampling, blast search and phylogenetic analysis

In order to reconstruct the timing of duplication that resulted in paralogous copies in *Actinidia chinensis, Roridula gorgonias *and *Saurauia zahlbruckneri*, we performed a BLAST search to look for all available *AGL6 *genes in the EST-database. The obtained sequences were combined with previously characterized *AGL6*-like sequences in a matrix (See additional File 2 - List of species used in the phylogenetic analysis with abbrevations and accession numbers). The short *AGL6*-sequences from *Asarum, Berberis, Anemone, Papaver, Citrus *and *Eschscholzia *were not included in this first matrix because they can be expected to artificially lower support values in resampling methods such as bootstrapping. Obtained nucleotide sequences were manually aligned using MacClade 4 [29], according to the reading frame of the conceptually translated amino acid sequences. The alignment starts at the RQVT-site in the MADS-domain and ends just before the stop codon. A multifasta alignment file can be found in the supplementary data (see additional File 3). After alignment, nucleotide sequences were analyzed with PAUP* 4b10 [30], Mr Bayes 3.1.2 [31], GARLI [32] and PHYML [33]. Paup* 4b10 [30] was used for parsimony bootstrap analysis. A Maximum Parsimony (MP) heuristic search was conducted using 1000 random addition sequences with TBR branch swapping and saving of multiple parsimonous trees (MulTrees on). Branch support values were obtained by nonparametric bootstrap analysis on 1000 pseudo-replicate data sets [34]. Parameters for the Bayesian analysis and maximum likelihood analysis were estimated using Modeltest 3.06 [35]. Modeltest selected the GTR+I+G substitution model using the Akaike Information criterion. MrBayes was run for 5 million generations where every 100 generations one tree was saved. The search reached stationarity around 50 000 generations. This number was considered the 'burnin period' and was excluded when the consensus phylogeny was constructed. PHYML [33] and GARLI [32] were used for maximum likelihood inference of the matrix. Confidence in the clades was estimated by the approximate likelihood ratio test method from Anisimova & Gascuel [36] and bootstrap analysis with 100 replicates. For the aLRT-tests, we used both the Chi2 probability and the more conservative SH-like test as branch support measures. The most likely tree was used and ML bootstrap values (>70) and BPP values (>90) were plotted on the three. In a second analysis, to identify the *AGL6*-sequences from *Asarum europaeum, Anemone nemorosa, Berberis julianae, Papaver somniferum, Eschscholzia californica *and *Citrus sinensis *as *bona fide AGL6 *sequences, we included these short sequences in the previous matrix and performed a second analysis using a similar strategy (See additional File 4 for the phylogenetic tree with bootstrap values from the likelihood analysis). To identify the newly identified *SEPALLATA3 *sequences as *SEP3 *homologs, we constructed a matrix with selected representatives from all *SEPALLATA*-subfamilies. We used *AGL6-*sequences as outgroup and constructed a neighbour Joining tree and performed parsimony bootstrap analyses with PAUP* 4b10 [30]. The resulting tree can be found in additional File 5. All included genes in this tree are listed in additional File 2.

### qRT-PCR quantification of gene expression

To examine the expression patterns of selected *AGL6 *and *SEPALLATA3 *genes using qRT-PCR, vegetative parts and floral organs of *Houttuynia cordata *and *Asarum europaeum *(Piperales, magnoliids)*, Eschscholzia californica, Papaver somniferum *and *Berberis julianae *(Ranunculales, eudicots), *Vitis vinifera *(Vitales, core eudicots), *Citrus sinensis *(Sapindales, rosids) and *Actinidia chinensis *(Ericales, asterids) were collected and frozen in liquid nitrogen. Floral material for *Asarum, Papaver, Eschscholzia, Berberis, Vitis, Citrus *and *Actinidia *were in the floral bud stage, except for *Houttuynia *where only mature flower material was available. The floral parts of *Eschscholzia, Papaver *and *Actinidia *were dissected from young mature flowers. RNA was extracted from each organ type separately using the Invisorb Spin Plant RNA kit (Invitek, Berlin, DE) or Trizol (Invitrogen, Carlsbad, US) and each RNA sample was DNase treated using TURBO DNA-free (Ambion, Austin, US). To verify the absence of gDNA in the total RNA, we used a PCR-reaction (40 cycles) with actin primers (data not shown). Based on this, we repeated the DNAse treatment for few samples. Total RNA was reverse transcribed into cDNA using AMV reverse transcriptase (Promega, Madison, US) and the included oligo-dT primer and random primers. Real-time PCR was performed on a StepOne Plus apparatus (Applied Biosystems, Forster City, US) using Fast SYBR Green Master Mix (Applied Biosystems, Forster City, US). Primers were constructed using the Primer Express software (Applied Biosystems, Forster City, US). The data presented here are the average of three technical replicates with standard error of the mean and two biological replicates are shown. All samples are normalized against *ACTIN *expression. Data analysis used the delta CT-method. For *Houttuynia cordata, Asarum europaeum *and *Berberis julianae, ACTIN *was cloned using a specific primer pair. New *ACTIN *sequences were deposited in Genbank [HM121983-HM121985]. The *SEPALLATA3 *sequences used are *VvMADS4 *for *Vitis vinifera *(XM_002275669), *CitMADS3 *for *Citrus sinensis *(AB218611), an EST-sequence from *Actinidia chinensis *(FG527965) identified as *SEP3*-like (see additional File 5). *EScaAGL9 *from *Eschscholzia californica *(AY850180) and *HcSEP3 *from *Houttuynia cordata *(AB089159). For all used primers see additional File 1: List of primer sequences used.

## Results

### Ancient duplications in the *AGL6 *lineage occurred at the base of the core eudicots and during monocot and magnoliid evolution

**Figure 1:**
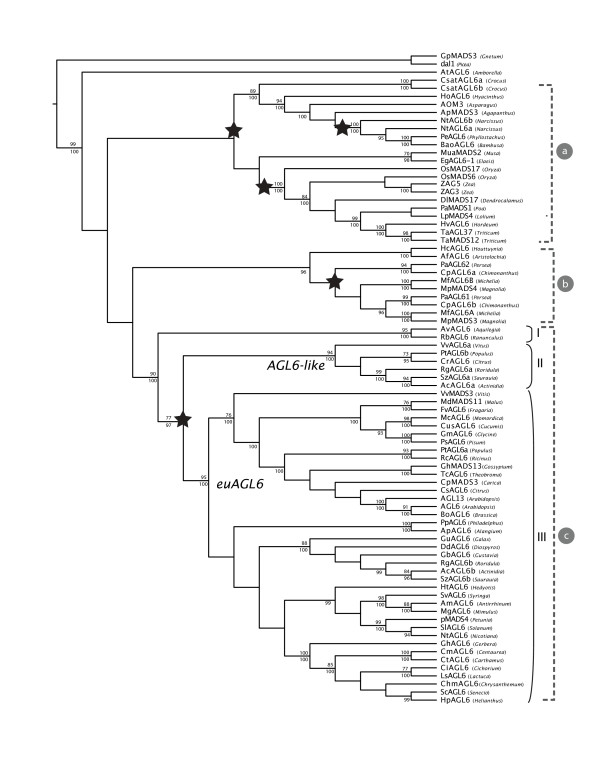
**Phylogeny of *AGL6 *genes in angiosperms, as inferred from maximum likelihood and Bayesian analysis**. Bayesian posterior probabilities (below) and bootstrap values from the likelihood analysis (above) are plotted on the tree. Black star indicates inferred duplication events. Included species, abbreviations and accession numbers are listed in additional file 2: List of species used in the phylogenetic analysis

For the reconstruction of the evolution of the *AGL6 *lineage, we combined previously identified *AGL6*-homologs with newly isolated and EST-derived *AGL6 *genes. The obtained data matrix contains two gymnosperm sequences and 79 angiosperm accessions and we analyzed this under maximum parsimony (MP) and maximum likelihood (ML) optimality criteria and in addition we used Bayesian posterior probability distribution estimation (BPP). We rooted the phylogeny by selecting gymnosperm *AGL6 *genes as outgroup. Because the maximum likelihood estimated topology and the Bayesian phylogram were identical, we present this tree as single most optimal phylogenetic estimate (Figure 1). Bayesian posterior probabilities and bootstrap values from the likelihood analysis are plotted on the tree as support measures for clades (Figure 1). MP bootstrap values, which were almost identical to ML bootstrap values, and branch support values from the aLRT-test, which again confirm the proposed hypothesis, are not shown.

While in previous phylogenetic analyses of the *AGL6 *lineage, eudicot sequences were sparsely sampled or the focus was on monocot representatives [16,17,23,37], the focus of this study is on the eudicot clade. Three major groups of *AGL6 *genes can be recognized in our estimate of phylogenetic relationships (Figure 1): a clade grouping monocot sequences (a); a clade grouping magnoliid sequences (b) and finally a large eudicot clade (c).

Within monocots (Figure 1a), gene relationships are not congruent with species relationships. Two Poales representatives (*PeAGL6 *from *Phyllostachys *and *BoAGL6 *from *Bambusa*) are nested strongly (ML 89 and BPP 100) in a clade grouping Liliales and Asparagales sequences. This is in turn sister to a clade containing all other Poales sequences (ML 100 and BP 100). The phylogeny thus indicates that during the divergence of monocots a first duplication event has occurred at least before the origin of Liliales [38]. However, no duplicated *AGL6 *genes were retrieved for any of the species in these groups, suggesting that paralogous copies may have been asymmetrically retained after duplication or remain to be identified. The fact that the Asparagales *Narcissus *sequence *NtAGL6a *groups more closely with Poales sequences *PeAGL6 *and *BoAGL6 *than with *NtAGL6b*, indicates a second ancient duplication tracing back to a time before the Asparagales branched off in monocot phylogeny. We would like to note here however, that sampling is particularly sparse to support the latter duplication and that although our analyses provide support, increased taxon sampling would improve accuracy for this inferred node [e.g. [39]]. In addition, our analyses show two duplicate *AGL6 *sequences for *Crocus *in Liliales (*CsatAGL6a, CsatAGL6b*) that we are unable to date more precisely. Finally we retrieve the ancient duplications in Poales shown by Reinheimer & Kellogg [23], leading to two copies in *Oryza sativa *(*OsMADS6 *and *OsMADS17*) and a second one leading to multiple copies in *Zea mays *(*ZAG3 *and *ZAG5*).

Within the magnoliid clade (Figure 1b), which is supported by a BPP-value of 96, both the representatives of Laurales (*Persea *and *Chimonanthus*) and Magnoliales (*Magnolia *and *Michelia*) possess multiple copies of *AGL6*. Although our analyses do not provide enough support for the timing of the duplication event, it probably occurred before the evolutionary origin leading to Magnoliales and Laurales [38]. However, another scenario involving two subsequent duplications can not be excluded.

Within the strongly supported eudicot group (Figure 1c, ML 90 and BPP 100), we recognize three supported clades: a basal eudicot clade with the Ranunculales representatives from *Aquilegia *(*AvAGL6*) and *Ranunculus *(*RbAGL6*) (Figure 1, I, ML 95 and BPP 100), a small core eudicot (Figure 1, II, ML 94 and BPP 100) and a large core eudicot clade (Figure 1, III, ML 95 and BPP 100). We propose to rename the large core eudicot clade the *euAGL6 *clade, similar to the eudicot duplications in the *AGAMOUS, APETALA3 *and *APETALA1 *lineage [5,28,40]. The small core eudicot clade will be referred to as the *AGL6-like *clade, as no functional characterization of any representative has been performed. In both *euAGL6 *and *AGL6-like *clades, rosid *AGL6 *sequences from *Vitis *(*VvAGL6a *and *VvMADS3*), *Populus *(*PtAGL6a *and *PtAGL6b*), *Citrus *(*CrAGL6 *and *CsAGL6*) and asterid *AGL6 *sequences from *Roridula *(*RgAGL6a *and *RgAGL6b*), *Saurauia *(*SzAGL6a *and *SzAGL6b*) and *Actinidia *(*AcAGL6a *and *AcAGL6b*) can be identified. Our data indicate that the duplication leading to these two paralogous clades occurred after the divergence of the basal eudicot Ranunculales clade and before the divergence of core eudicots [38]. This scenario is supported by a BPP-value of 97 and a bootstrap value of 77. Although duplications frequently occur in almost all MADS-box gene lineages, this ancient duplication event was not identified in previous analyses of the *AGL6 *lineage. The *AGL6-like *clade (Figure 1, I) groups only few species, despite our targeted efforts to identify more sequences belonging to this clade in sequence databases. Within the *euAGL6 *clade (Figure 1, II), no disruptions of gene to species relationships indicate later additional duplications. As expected from our knowledge of evolutionary relationships within angiosperms, a rosid (ML 76 and BPP 100) and an asterid clade are recognized.

### Expression patterns of duplicated eudicot *AGL6 *genes have diverged and differ from *SEP3 *genes

**Figure 2:**
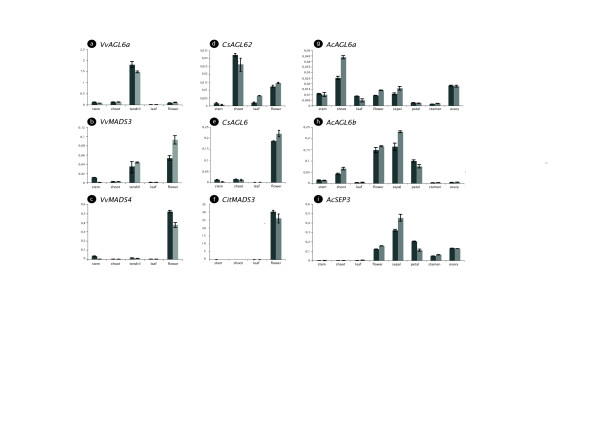
**Expression of duplicated *AGL6*- and *SEPALLATA3*-genes in core eudicot species using qRT-PCR**. *AGL6 *and *SEP3 *qRT-PCR products from *Vitis vinifera *(a-c), *Citrus sinensis *(d-f) and *Actinidia chinensis *(g-i) are shown. Relative expression to *ACTIN *is shown using the delta Ct method. Floral material for *Vitis, Citrus *and *Actinidia *were in the floral bud stage. The floral parts of *Actinidia *were dissected from young mature flowers.

Information on expression patterns of *AGL6 *genes for eudicot representatives is even more limited as compared to monocots [23] and magnoliids [4]. Until now, one rosid species (*Arabidopsis thaliana*) [13,14], one basal eudicot species (*Vitis vinifera*) [41] and one asterid species (*Petunia hybrida*) [17,42] have been investigated for their *AGL6 *expression patterns. Because our analyses indicate an ancient duplication at the base of the core eudicots with resulting paralogous copies, we studied the expression patterns of these duplicated *AGL6 *genes in *Vitis, Citrus *and *Actinidia *using qRT-PCR (Figure 2) in comparison to expression of *SEP3 *mRNA's [17].

In *Vitis vinifera*, expression of the two *AGL6 *genes differs markedly: while the *AGL6*-homolog from the *AGL6-like *clade (*VvAGL6a*, Figure 2a) is predominantly expressed in tendrils, expression of its paralog (*VvMADS3*, Figure 2b) is both in tendrils and flowers. Previously, it was shown that the expression of *VvMADS3 *was restricted to the floral parts of the plant and the expression in tendrils we show here was not detected [41]. This could be explained by the difference of specific sampling of tissues between both studies. The *SEP3 *gene (*VvMADS4*, Figure 2c) is most strongly expressed in the flower and confirms the observations of a previous study in *Vitis *[43]. Thus, both *VvMADS3 *and *VvMADS4 *are expressed in the flower, while *VvAGL6a *is most strongly expressed in tendrils. In contrast to *VvMADS4*, both *AGL6 *genes are also expressed in tendrils. Although tendrils would seem vegetative structures in nature, a putative homology of tendrils and reproductive shoots has been proposed, partly on the basis of *AP1 *and *FUL *expression [44].

For the rosid genus *Citrus*, we also found one paralog belonging to the *AGL6-like *lineage (*CrAGL6 *and *CsAGL62*) and one paralog grouping with the *euAGL6 *clade (*CsAGL6*). The expression patterns of both genes were examined in vegetative and floral parts of *Citrus sinensis*. Again, the expression patterns between the duplicated *AGL6 *genes differ significantly. While the *AGL6-like *gene (*CsAGL62*, Figure 2d) is expressed both in the vegetative shoot and the flower of *C. sinensis*, the *euAGL6 *gene (*CsAGL6*, Figure 2e) is predominantly expressed in the flower. We compared this to the expression pattern of a *SEP3*-homolog in *Citrus, CitMADS3 *(Figure 2f) [45]. Similar to the results of Endo *et al. *[45], the *SEP3*-homolog is strongly expressed in floral tissues. So both *CitMADS3 *and *CsAGL6 *are expressed in the flower, while *CsAGL62 *is expressed predominantly in the vegetative shoot.

Also, in *Actinidia chinensis*, the two *AGL6*-paralogs have acquired different expression patterns. *AcAGL6a*, which belongs to the *AGL6-like *clade is expressed both in floral parts and in all vegetative tissue samples (Figure 2g), but predominantly in the vegetative shoot. The *euAGL6 *gene in *Actinidia, AcAGL6b *is also expressed in both vegetative and floral tissues (sepals and petals), but predominantly in floral tissue (Figure 2h). The expression pattern in floral tissues is rather similar to the expression of the ortholog of *AcAGL6a *in *Petunia, pMADS4*, of which the expression is confined to floral organs [17,42]. According to expectations, the *SEP3 *gene in *Actinidia *is only expresssed in floral parts (*AcSEP3*, Figure 2i). The expression of *pMADS4 *in the floral organs of *Petunia*, which is mostly in the petal and the ovary, is different from the expression of the *AGL6 *genes in *Actinidia*. While the *AGL6*-like homolog is expressed in sepals and ovaries (*AcAGL6a*, Figure 2g), the *euAGL6 *copy is mainly expressed in sepals and petals (*AcAGL6b*, Figure 2h).

It is obvious from these results that the duplicated *AGL6 *genes in *Vitis, Citrus *and *Actinidia *have acquired different expression patterns after duplication. While in *Vitis *and *Actinidia*, the paralog from the *AGL6-like *clade is strongly expressed in tendrils (*Vitis*) or in the vegetative shoot (*Actinidia*), the *euAGL6 *paralog is expressed in tendrils and floral tissues for *Vitis *and shoot and floral tissues for *Actinidia*. Therefore, *AGL6 *expression in *Vitis *and *Actinidia *is different from *SEP3 *genes, which are predominantly expressed in floral tissues. In the rosid species *Citrus*, the situation is different; the gene belonging to the *AGL6-like *clade is expressed in both vegetative and floral parts while the *euAGL6 *gene has an expression restricted to floral tissues, similar to expression of the *SEP3 *gene. Thus, while *SEP3*-expression is always limited to floral tissues, *euAGL6 *genes are also predominantly expressed in floral tissues with some vegetative expression. Contrary, expression levels of *AGL6*-*like *genes are highest in the vegetative shoots and/or tendrils.

### Vegetative expression was lost in angiosperms and regained in core eudicots

**Figure 3:**
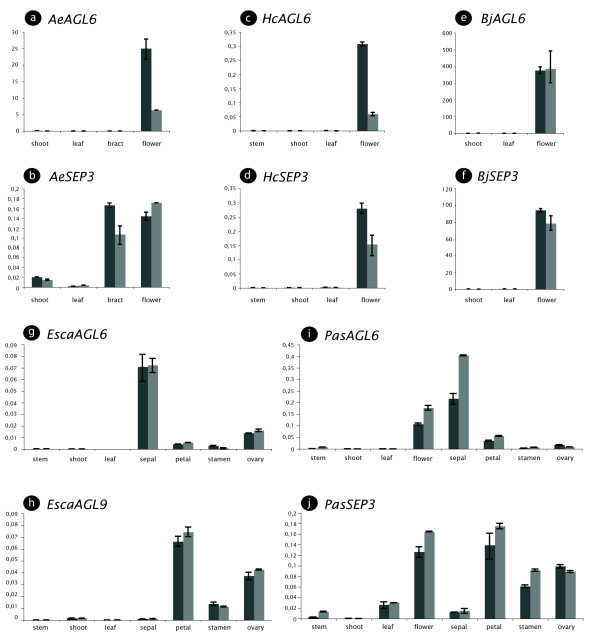
**Expression of *AGL6*- and *SEPALLATA3*-representatives in selected magnoliid and Ranunculales species using qRT-PCR**. *AGL6*-like and *SEP3*-like qRT-PCR products from *Asarum europaeum *(a-b), *Houttuynia cordata *(c-d), *Berberis julianae *(e-f), *Eschscholzia californica *(g-h) and *Papaver somniferum *(i-j) are shown. Relative expression to *ACTIN *is shown using the delta Ct method. Floral material for *Asarum, Berberis, Papaver *and *Eschscholzia *were in the floral bud stage, except for *Houttuynia *where only mature flower material was available. The floral parts of *Papaver *and *Eschscholzia *were dissected from young mature flowers.

Our phylogenetic studies reveal that several plant lineages can be expected to harbor more than one *AGL6 *representative in their genome and that these paralogous sequences can be of ancient origin. Because gene duplicates in MIKC type MADS-box genes have been shown repeatedly to sub- or neofunctionalize, with gene expression divergence as an observable outcome [46-48], we attempted to infer ancestral *AGL6 *expression patterns in flowering plants. This should help to phylogenetically root and trace the evolution of gene function in this subfamily of MADS-box transcription factors. Previous expression patterns of *AGL6 *representatives in magnoliids [4,49], Nymphaeles and Proteales [22], non-grass monocots [24,25,37,50] and Poaceae [23] did not find expression in vegetative tissues. However, we showed that some duplicated eudicot *AGL6 *genes are expressed in vegetative tissues (cfr supra) and in gymnosperms an *AGL6*-homolog, *PrMADS3*, was detected in the group of cells initiating needle primordia in vegetative buds from *Pinus radiata *[20]. To investigate when expression in vegetative tissues may have originated, we quantitatively compared the expression level of *AGL6 *in vegetative and reproductive tissue samples of several species belonging to the magnoliids and Ranunculales, two clades that originated before the divergence of the core eudicot clade. Again here, we compared the expression of *AGL6 *to that of *SEP3 *mRNA's in both vegetative and reproductive tissue samples (Figure 3).

We selected two representatives from the Piperales (magnoliid clade), *Asarum europaeum *and *Houttuynia cordata *(Figure 3a-d). In both species, expression of the *AGL6*-representatives, *AeAGL6 *and *HcAGL6*, is restricted to floral tissue (Figure 3a,c). In contrast to *AeAGL6*, the *SEP3*-homolog from *Asarum europaeum *(*AeSEP3*) is not only expressed in floral tissue, but also in the bracts enveloping the flower (Figure 3b). In *Houttuynia cordata*, expression of a *SEP3 *gene (*HcSEP3*) is similar to that of a *AGL6 *gene (Fig 3d). This is in agreement with the observations for *Persea americana *and *Magnolia grandiflora*, for which no vegetative expression of *AGL6 *representatives was detected [4,49].

We next selected three representatives from the Ranunculales, which is close to the ancient duplication event at the base of the core eudicots, to check for expression in vegetative tissue. Expression of *AGL6 *representatives was investigated in reproductive and vegetative tissue samples from *Eschscholzia californica *and *Papaver somniferum *(Papaveraceae) and *Berberis julianae *(Berberidaceae) using qRT-PCR (Figure 3e-j). In *Berberis julianae*, we found that *AGL6 *is solely expressed in the floral tissue (*BjAGL6*, Figure 3e), similar to *SEP3 *expression (*BjSEP3*, Figure 3f). Also for both representatives of the Papaveraceae, *Eschscholzia californica *(*EscaAGL6*, Figure 3g) and *Papaver somniferum *(*PasAGL6*, Figure 3i), *AGL6 *is restricted to floral parts. The *SEP3 *genes from these two species are also predominantly expressed in floral tissues (*EscaAGL9 *and *PasSEP3*, Figure 3h,j), yet low expression of *PasSEP3 *can be found in leaves and even stem (Figure 3j). Within the flower, expression of both *EscaAGL6 *and *PasAGL6 *is mainly maintained in the sepals with low expression in the ovary for *EscaAGL6 *and in the petals for *PasAGL6 *(Figure 3g,j). In contrast, the expression of *EscaAGL9 *and *PasSEP3 *(Figure 3h,j) is in all floral organs, except in sepals.

The detected vegetative expression pattern in gymnosperms and several core eudicots and the absence in monocots, magnoliids and Ranunculales suggests that expression in the shoot apical meristem or more broadly the meristem and young leaves (entire shoot) was lost around the origin of the angiosperm clade and regained after the duplication at the base of the core eudicots.

## Discussion

### The *AGL6 *gene phylogeny is not congruent with the corresponding species phylogeny

**Figure 4:**
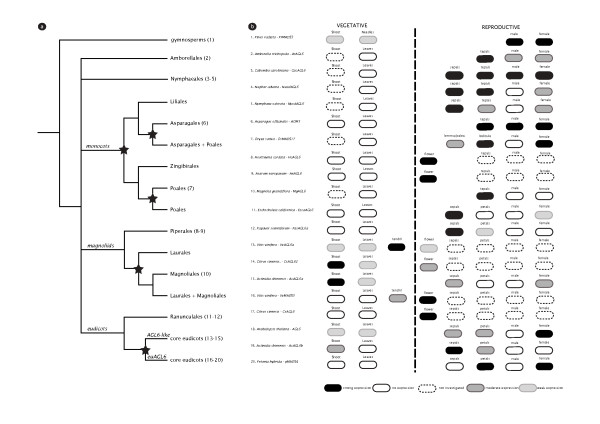
**Evolution of the *AGL6 *lineage and *AGL6 *expression patterns in gymno- and angiosperms**. (a) Summary of the results of the phylogenetic analysis. Black stars indicate inferred duplication events. (b) Expression of selected representatives in both vegetative and reproductive structures is shown: strong expression (black), moderate expression (gray), weak expression (light gray), absence of expression (white) or not investigated expression (white with dashed line). Expression data were taken from Mouradov *et al. *[20], Kim *et al. *[4], Ohmori *et al. *[16], Reinheimer & Kellogg [23], Rijpkema *et al. *[17], Schauer *et al. *[14], Yoo *et al. *[22] and this study.

Our phylogenetic estimates indicate five ancient angiosperm duplications in the molecular evolution of the *AGL6 *lineage of which four were not detected in previous analyses [14,16,17,24,37] (summarized in Figure 4a). Two of these duplications occurred during monocot evolution: a first one before the Liliales branched off and a second one before Asparagales branched off. A third ancient duplication could be demonstrated during magnoliid diversification. Probably least expected, a fourth duplication event occurred before the origin of the core eudicot clade resulting in *euAGL6 *and *AGL6-like *genes. In contrast to previous phylogenetic analyses, it required the inclusion of sequences from basal asterids and EST-derived eudicot *AGL6 *sequences to generate support for the core eudicot duplication, illustrating that the pattern of gene loss or divergence in expression patterns of MADS-box genes requires elaborate sequence and taxon sampling to reveal the molecular evolutionary history of this gene family.

As is the case for the *APETALA1, APETALA3, AGAMOUS *and *SEPALLATA *gene lineages [5,28,40,51-53], the *AGL6 *lineage underwent a duplication that can be traced back to the origin of the core eudicot clade. It has been suggested that the resulting paralogous sequences could be the result of a whole genome duplication in an ancestor of core eudicots after which transcription factors have been preferentially retained [5,54]. Yet, an alternative scenario involving multiple independent duplication events has also been suggested as an explanation for the resulting duplicate lineages and both scenarios are not mutually exclusive [53]. Our results indicate that the *euAGL6 *and *AGL6-like *sequences were likely retained early after a genome duplication around the origin of the core eudicots. The phylogeny further shows that the *euAGL6 *lineage is represented by many more sequences than the *AGL6-like *lineage. One possible explanation for this observation is that the *AGL6-like *lineage was lost more frequently in later speciation events, resulting in a small core eudicot clade with few paralogous sequences. This is illustrated by the absence of the *AGL6*-like lineage in the genome of *Arabidopsis thaliana *as both *AGL6 *and *AGL13 *from *Arabidopsis *belong to the *euAGL6 *clade. Alternatively, the fact that one clade is much smaller than the other could equally well indicate that members of the smaller clade are expressed in a more restricted or different temporal or spatial domain not sampled by EST sequencing or missed in targeted cloning efforts. Although we are currently unable to attribute functions or diversification in function of the duplicated core eudicot *AGL6*-like sequences in this study, expression divergence between the paralogous copies is strong, suggesting significant evolution in the transcriptional regulation of these genes.

### Maintenance in male organs appears ancestral in flowering plants, but was frequently lost and vegetative expression was gained in core eudicots

Ancestral expression patterns of *AGL6 *in reproductive structures were previously reconstructed in Schauer *et al. *[14] and Reinheimer & Kellogg [23]. It was suggested that expression of the *AGL6 *lineage in floral meristems has been conserved since angiosperm diversification [23]. In angiosperms, the inferred ancestral expression included both male and female reproductive tissues and male-specific expression was lost later in evolution [14]. Our updated summary of *AGL6 *expression patterns confirms this inference and adds to the complexity of the evolution of gene expression in the *AGL6 *lineage (Figure 4b). A recent study that included Nymphaeales *AGL6 *genes shows that expression of *AGL6 *in *Nymphaea *or *Nuphar *is absent from stamens, while out of three *AGL6 *paralogs from *Cabomba*, only *CacaAGL6-2 *is detected in male organs [22]. Also in grasses, expression in male organs was lost [23]. In basal eudicots, *AGL6 *expression in *Nelumbo *(Proteales) is weak in stamens [22] and in our study we find that in both *Eschscholzia *and *Papaver*, expression is not maintained in stamens relative to other organs. In core eudicots, for neither the *euAGL6 *or the *AGL6-like *lineages, we detected expression in stamens. Together these results indicate that although originally in gymnosperms and angiosperms expression was likely maintained in both male and female organs, from the early diversification of flowering plants, expression maintenance in male organs disappeared in most lineages. We have to mention that the expression levels summarized in Figure 4b may depend on the developmental stage of the (floral) tissues sampled, which in turn may influence a comprehensive summary of the data currently available.

We also investigated the expression in developing vegetative tissues. Consistent with previous observations, we find that the expression of *AGL6 *genes in angiosperms is confined to reproductive tissues before the origin of core eudicots (Figure 4b). After the origin of the core eudicot *euAGL6 *and *AGL6-like *lineages, these lineages gained expression in vegetative tissues. Yet in *Actinidia *(Ericales) and *Citrus *(Rutales), expression of *AGL6-like *is stronger in vegetative shoot tissue than in floral bud tissue. Also in *Vitis *(Vitales), we measure similar expression levels of the *AGL6-like *gene in vegetative shoots and flower tissue and intriguingly, expression is most pronounced in tendrils. In contrast, the *euAGL6 *lineage has overall retained its expression domain confined to floral tissues and moderate expression is also observed for vegetative tissues of *Actinidia*. For several other members of this lineage, expression in vegetative tissues is either confined to a restricted domain (e.g. *Arabidopsis*) or not detected at all (e.g. *Petunia*), suggesting that the *euAGL6 *lineage overall performs roles confined to reproductive development, though additional roles in vegetative development cannot be excluded for some species. Furthermore, functional diversification of the *euAGL6 *and *AGL6-like *lineages is not evident from characteristic motifs found at the C-terminal end of the proteins, as has been observed for several other MADS-box gene lineages that originated before the origin of core eudicots [5,28,40,51-53]. Rather, the phylogenetic signal that separates the two lineages is distributed throughout their sequences. Overall, our and previous data suggest that the expression in vegetative tissues of the *AGL6 *lineage is novel in core eudicots. More extensive studies are needed to determine when relative vegetative expression in core eudicots was acquired and how *euAGL6 *and *AGL6-like *lineages parsed an ancestral *AGL6 *function.

### A putative role of *AGL6 *genes in vegetative development

In *Arabidopsis*, both *AGL6 *and *AGL13 *paralogs belong to the *euAGL6 *lineage, and no *AGL6-like *sequence has been annotated, suggesting that the *AGL6-like *lineage was lost in the evolution to *Arabidopsis*. Yet data from *Arabidopsis *may be indicative of multiple roles for the *AGL6 *subfamily. Several lines of evidence have now established a (redundant) function for the *AGL6 *lineage in reproductive development [16-18]. Recent two-hybrid and three-hybrid assays have indicated that the *Arabidopsis *AGL6 protein interacts with other MADS-domain proteins known to perform roles restricted to reproductive development such as AGAMOUS, APETALA1, SHATTERPROOF2 and SEPALLATA3 [11,55]. The function of *PhAGL6 *in *Petunia *was indeed only revealed in double mutant combinations with *PhSEP3 *[17] and similarly the moderate *mfo1 *phenotype in rice became severe in combination with a mutation in the *SEP*-like gene *LHS1 *[16]. In a third example, mutation of the maize *AGL6 *homolog *bearded ear *has a number of phenotypes shared with mutation in the maize *AGAMOUS *homolog *ZEA AGAMOUS 3 *and the combination of *bde *and *zag3 *results in the complete conversion of floral meristems into branch-like meristems [18].

In addition to the role in reproductive development, we have observed that members of the *AGL6*-like lineage acquired expression in vegetative shoot tissue. We speculate that this expression could be related to a function in flowering time. Several other observations can be interpreted to point in this direction. The protein interaction profile is again indicative: *Arabidopsis *AGL6 interacts with SHORT VEGETATIVE PHASE, SUPRESSOR OF OVEREXPRESSION OF CONSTANS1 and FRUITFUL [11]. These proteins have a well-described regulatory role in the transition to flowering, a process regulated in the leaf and shoot apical meristem. *FUL *and *SOC1 *are indeed expressed during vegetative development and their expression is apparent in the shoot apical meristem upon floral induction [56-58]. An additional combined function in the cambium has recently been described for these proteins [59]. Similar to *FUL *and *SOC1, AGL13 *appears to be expressed in the vasculature of *Arabidopsis*, leaving the possibility that *AGL6 *genes are regulating processes in the cambium together with *FUL *and *SOC1*. The idea that members of the *AGL6 *lineage, possibly in combination with *FUL*, may perform an additional role related to the phase transition of the adult shoot into the reproductive developmental program is further suggested by the fact that both *Arabidopsis AGL6 *and *FUL *are strongly upregulated in a *microRNA172a *mutant background [60]. MicroRNA172 targets members of the APETALA2 family of transcription factors such as SCHLAFMÜTZE, which in turn act as repressors of flowering [61]. Such a regulatory role for *AGL6 *or *AGL13 *might have gone unnoticed thus far as a result of redundancy with other factors. A recent study similarly suggests a function related to flowering time for *Arabidopsis AGL6 *[15]. Constitutive expression or fusion to the EAR repressor domain resulted in early flowering and in such lines, expression levels of known regulators of flowering time were modified. Earlier ectopic expression experiments of *AGL6 *genes from several monocot species [24-26] similarly resulted in early flowering *Arabidopsis *transformants. However, in another experiment, the effect on flowering time was absent when *AGL6 *was expressed under its native promoter, which is unexpected if *AGL6 *would play a role in controlling *Arabidopsis *flowering time [15]. A similar series of experiments should be performed for its paralog, *AGL13*, to be able to extend conclusions to the entire *euAGL6 *lineage in *Arabidopsis*. The shoot expression observed in this study contributes to the idea that the *AGL6 *lineage may have regained a function in regulating aspects of the floral transition in core eudicots. This should be functionally investigated in a species that retained both *euAGL6 *and *AGL6-like *gene lineages.

## Conclusions

Phylogenetic inference of the *AGL6 *subfamily of MADS-box transcription factors indicates that four ancient duplications occurred during the evolution of this lineage. As is the case for other MADS-box gene lineages, one of these duplications occurred at the base of the core eudicots and resulted in *euAGL6 *and *AGL6-like *gene clades of which the representatives show strong expression divergence. Thus far, *AGL6 *gene expression was observed only in reproductive structures, but our analyses indicate additional expression in vegetative shoots after the core eudicot duplication. Though speculative, this may indicate that *AGL6 *genes perform a function in the developmental transitions of shoots, in addition to their function in the reproductive structures.

## Authors' contributions

TV and KG layed out the work performed. TV performed experiments and TV and KG drafted the manuscript. DV provided a *SEP3*-alignment. AB provided material of *Eschscholzia californica *and critically revised the manuscript. SM provided additional ideas for the discussion of data in the manuscript. All authors read and approved the final manuscript.

## Supplementary Material

Additional file 1 List of primer sequences used in the expression analysis. Click here for file

Additional file 2 List of species used in the phylogenetic analysis of *AGL6 *and *SEP3 *with abbrevations and accession numbers (black stars indicate EST-data). Click here for file

Additional file 3 **Multifasta alignment file of the *AGL6*-matrix**. Click here for file

Additional file 4 Identification of new *AGL6 *representatives from *Asarum europaeum, Anemone nemorosa, Berberis julianae, Papaver somniferum, Eschscholzia californica, Anemone nemorosa and Citrus sinensis *(highlighted in gray). Bootstrap values from the likelihood analysis are plotted on the most likely tree as support meausures. Click here for file

Additional file 5 Identification of new *SEP3-*representatives from *Asarum europaeum, Berberis julianae, Anemone nemorosa, Papaver somniferum *and *Actinidia chinensis*. Neighbour-Joining tree with bootstrap values (above branches) and bootstrap values from parsimony analysis (below branches). Newly identified *SEP3*-sequences are highlighted in gray. Click here for file
