# Supplementary figures and images for: Expression divergence of the AGL6 MADS domain transcription factor lineage after a core eudicot duplication suggests functional diversification
Source: BMC Plant Biol. 2010 Jul 15;10:148. doi: 10.1186/1471-2229-10-148 (PMC3095293; doi:10.1186/1471-2229-10-148)

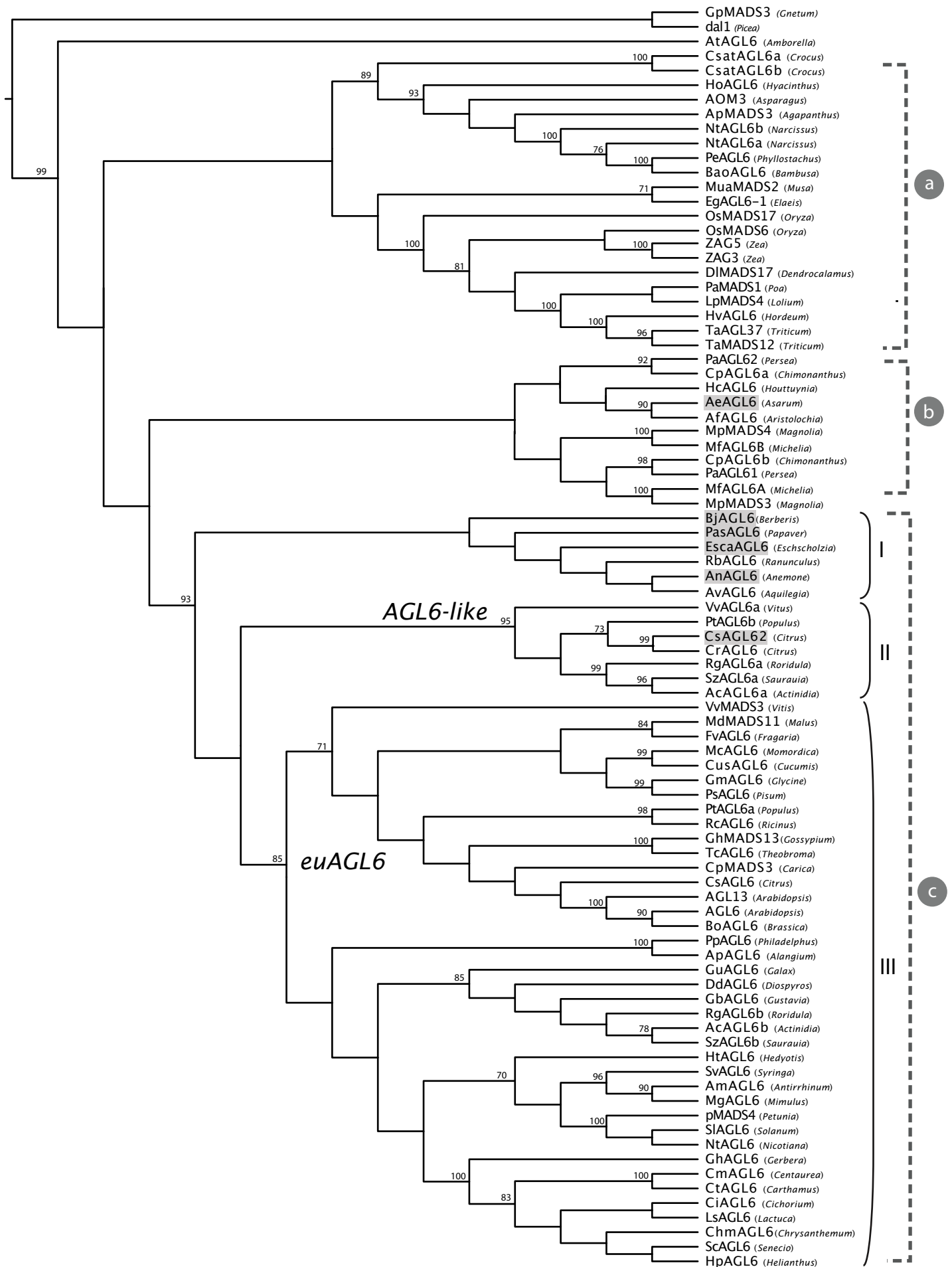

Supplement: Additional file 4 — Identification of new AGL6 representatives from Asarum europaeum, Anemone nemorosa, Berberis julianae, Papaver somniferum, Eschscholzia californica, Anemone nemorosa and Citrus sinensis (highlighted in gray). Bootstrap values from the likelihood analysis are plotted on the most likely tree as support meausures. [file 1471-2229-10-148-S4.PDF]

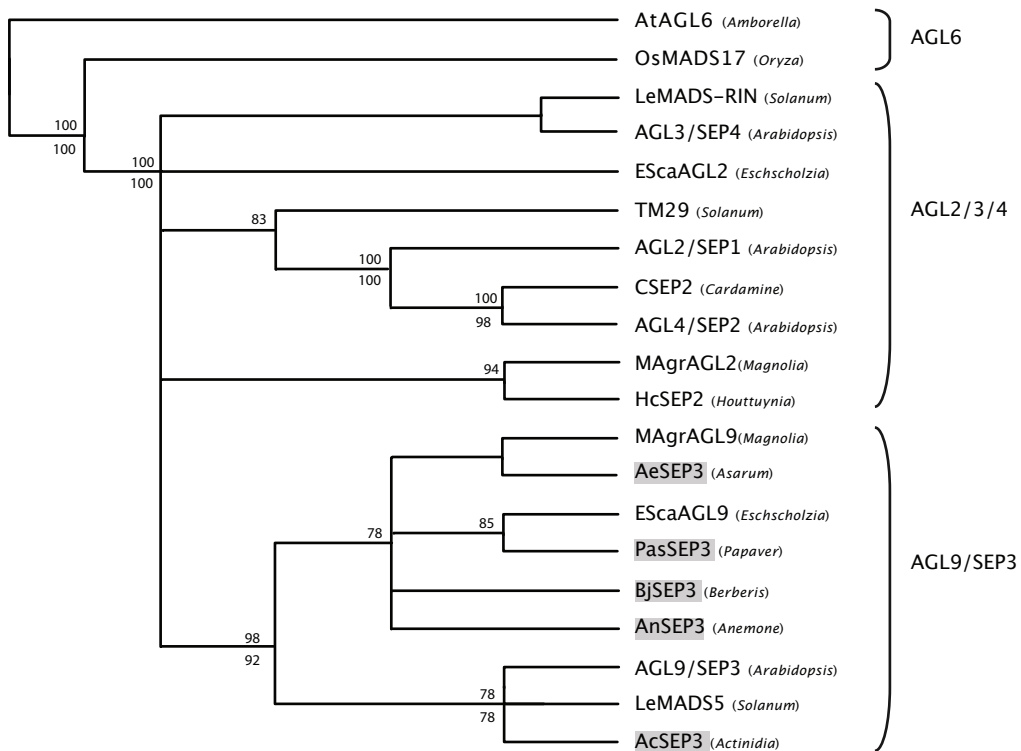

Supplement: Additional file 5 — Identification of new SEP3-representatives from Asarum europaeum, Berberis julianae, Anemone nemorosa, Papaver somniferum and Actinidia chinensis. Neighbour-Joining tree with bootstrap values (above branches) and bootstrap values from parsimony analysis (below branches). Newly identified SEP3-sequences are highlighted in gray. [file 1471-2229-10-148-S5.PDF]
